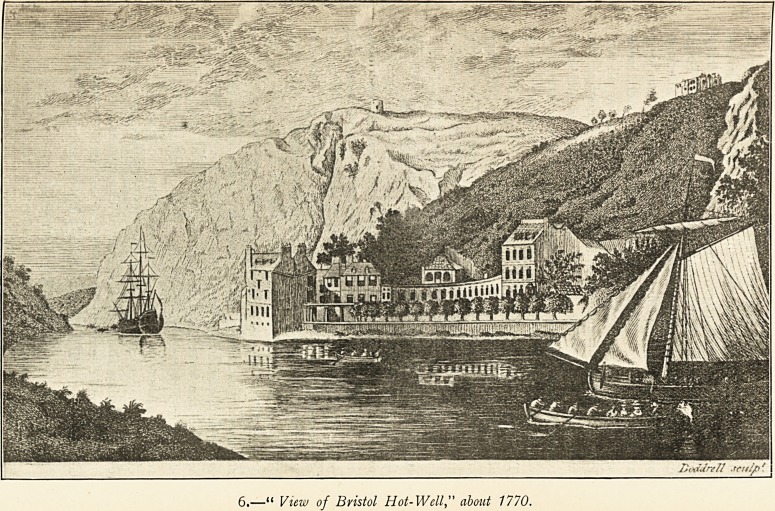# The Reputation of the Hotwells (Bristol) as a Health-Resort

**Published:** 1902-03

**Authors:** L. M. Griffiths


The Bristol

Medico-Chirurgical Journal.

MARCH, 1902.

### THE REPUTATION OF THE HOTWELLS (BRISTOL) AS A HEALTH-RESORT.

BY

L. M. Griffiths, M.R.C.S. Eng.

When I was Honorary Librarian of the Bristol Medical Library, I read before the Library Association a paper entitled "Some Things of General Interest in the Bristol Medical Library."[1] This contained much about the social and literary life of the Hotwells when it was a fashionable health-resort. Some passages from that paper are here repeated, and at the request of the Editor of this *Journal* I have added from the books to which reference was then made some matter which could not be mentioned when I read the paper before the members of the Library Association.

The reputation of the Hotwell water goes back to the middle of the fifteenth century, when it was mentioned by William Wyrcestre, who was a Bristol man. From the beginning of the seventeenth century the virtues of the Hotwell water

[1] *Library Association Record*, June and July, 1901.

have been proclaimed by various authorities, medical and otherwise. Dr. Tobias Venner of Bath, whose work,[1] published first in 1620, reached a third edition in 1650, said that the water "is in great request and use against the Stone." Venner had studied the properties and action of the water with much minuteness. In reference to the former, he states that "whatsoever minerals shall lie hid in the passages of this water, it is sufficient, that it partakes of two so good as Sulphur and Niter, and that in such a mixture, as it makes it to be of an excellent temper, and medicinable facultie in potable uses for divers cases."[2] He considered that the reputation of the water was certain to suffer because patients were resorting to it for objects other than "the drinking of it against the Stone," and that it should only be taken under the advice of a "judicious Physician." Without such guidance "the ill and preposterous use thereof will weaken the stomack, subvert the liver, annoy the head and brest, occasion Cramps, paine in the joynts, breed crudities, rheumes, coughs, Cachexies, the Dropsie itselfe, and Consumption."[3] Further emphasis is laid on the necessity of skilful counsel when sick folk were taking the water. Admitting its "excellent faculties," he regarded it as "very effectuall against the burning heat of the stomack, inflammations of the liver and reines, and adustion[4] of the humors, being taken with fine Sugar in this proportion, as a dram of Sugar or there about to a pint of the Water."[5] Venner seems to have had little difficulty in diagnosing "purulent ulcers of the kidneys and bladder, and ulcerations of the intestines;" and in these conditions the water, taken with the necessary expert caution, was good. He gives ten rules for its use in cases of vesical calculus. These refer—

(1) To the preparation of the patient, whose body was to be "exquisitely purged";

[1] *Via Recta ad Vitam Longam*, of the 1638 edition of which "A Censvre concerning the Water of Saint Vincents Rocks neere Bristoll" formed part. Of course, censure was used with the meaning which it had not then lost of "opinion" merely. Cf. *The Winter's Tale*, II., i. 36.

[2] P. 334. [3] P. 335.

[4] [This word is now obsolete. It signified a burning, and is allied to "combustion."]

(2) To the taking of the water fasting;

(3) and (4) To the quantity to be drunk and the duration of the treatment, both of which matters were to be left to the judgment of the physician in charge of the case;

(5) To the temperature at which the water was to be taken, bearing in mind that it was not to lose the heat[1] which it possessed at the spring, and if it were impossible to take it in that way it was to be kept in a stone jug, which was to be heated in a kettle of hot water until it was as hot as the patient could take it. If it was kept it was likely to lose "somewhat of its sulphurous, but not anything of its nitrous quality";

(6) To the time of year when it might be used most advantageously, that being from the beginning of May to the middle of September;

(7) To the diet of the patient whilst undergoing treatment. This should be "slender," and dinner was not to be taken "till the greater part of the Water be avoyded, and the supper must bee alwaies lesse than the dinner, that the Stomack may be the next morning emptie for receiving of the Water againe";[2]

(8) To the necessity of not allowing the water to "abide in the body after the use of it";

(9) To the ages of the patients, who could be fit recipients of the water. It was to be given cautiously to children under twelve years of age, and not at all to those who had "entered within the limits of old age, because it will abreviate their life, *calorem innatum extinguendo*";

(10) To the idiosyncrasies of the patients, for on no account was it to be administered to "such as by reason of the smalnesse and straightnesse of their veines, cannot excrete and passe it away by urine." Neither was it to be given to "such as have cold stomacks, weake livers, feeble braines, and subject unto Rheumes; in a word, not to phlegmatick, nor to any that abound with crudities, or have a cold and moist habit of body: for in all such it will soone infringe the naturall heat, breed Rheumes, annoy the brest, occasion Cramps, and divers other infirmities."[3]

Venner concludes his directions by again dwelling upon the importance of "the advice and presence of a judicious Physician" in all cases. He thought little was to be gained from the external application of the water, although it "may somewhat asswage the Itch, mundifie[4] and palliat old Sores." It will be seen from these extracts that Venner was exceed-

[1] According to later observers, who had the Fahrenheit thermometer, this was about 76°. [2] Pp. 339-40. [3] P. 341.

[4] ["**Mundifie.** To cleanse." Cockeram's *English Dictionarie*. Fifth Edition. 1637.]

ingly careful about details, and probably on that account was a successful practitioner. He is no less precise in his directions about tobacco, which he added in "A Briefe and Accurate Treatise concerning the taking of the Fume of Tobacco, which very many, in these dayes, doe too too licenciously use. In which, the immoderate, irregular, and unseasonable use thereof is reprehended, and the true nature and best manner of using it, perspicuously demonstrated." He approved it "as necessary and profitable for the rheumatick, and such as are of a cold and moist constitution, and in cold and moist seasons, so as it be taken in congruent manner, that is, both moderately and seasonably."[1] Venner evidently felt keenly the competition of the irregular practitioner and apothecary; for, speaking of those who thought the combination of tobacco and sack might often render the services of the physician unnecessary, he deplored the fact that "very many of our people, in their sicknesse, expose their bodies to bee corrupted, I cannot say cured, to ignorant usurping Poticaries, and other base illiterated Empericks, who are (contrary to the Lawes) everywhere permitted to exercise Physick, to the dishonour of God, disgrace to the Faculty, hurt of our people, and shame of our Nation."[2] His love of detail led him to give those who affected tobacco ten precepts in the use thereof. In these and in his directions concerning the Hotwell water one cannot fail to be struck with the sublime confidence he had in his own judgment, and this must have made him a very acceptable attendant to patients who especially abhor any uncertainty in the opinions and practice of their doctor. Venner was buried in Bath Abbey, where is erected a tablet with a very eulogistic inscription, to the Latinity of which Dr. Thomas Guidott takes exception in some exceedingly quaint criticism.[3]

Dr. Thomas Johnson, in *Mercurius Botanicus*, published in 1634, stated that the water was of repute both inwardly and outwardly. In 1662, in Fuller's *Worthies of England*, it is declared

[1] P. 349. [2] P. 354.

[3] "The Lives and Characters of the Physicians of Bath," in *A Discourse of Bath, and the Hot Waters There. Also, Some Enquiries into the Nature of the Water of St. Vincent's Rock, near Bristol, and that of Castle-Cary.* Second Edition, 1725, pp. 189-91.

that "St. *Vincents Well* is sovereign, for *Sores and Sicknesses*, to be *washt* in, or drunk of," and the statement is made that "experience proveth that Beer brewed thereof is wholesome against the *Spleen;*" and it is related that Dr. Samuel Ward, of Sidney College, Cambridge, who "was afflicted with that malady, was prescribed the constant drinking thereof, though it was costly to bring it thorough the *Severn*, and *narrow Seas* to *Lin*, and thence by the River to *Cambridge*."[1] The Hotwells received its first royal visitor in 1667, when Queen Catherine, the wife of Charles II., after dinner in Small Street, drove to see the Avon Gorge and drank some of the water.[2]

A doctor who is known as Claromontius, which was probably a Latin version of an English name, visited the Hotwells, and in 1672 gave his experience of the water in a book entitled *De Aere, Locis, et Aquis terræ Angliæ.* Although he records its good effect generally in "the Gravel, and Obstructions of the Intestines," he did not get much personal benefit; the Waters "made him puke, and did not pass as they should do by Urine," and if this result did occur, there was a danger that they might "gripe the Bowels and cause Ruptures."[3] Verily, in the good old times the Hotwell Water was not a thing with which one could trifle!

Dr. Thomas Guidott, of Bath, writing in 1676, considered that the Hotwell Water "may be as effectual as *Tunbridge* Waters, in any Diseases that Water is proper for."[4] He published the results of an examination of the water which he had made with the help of Mr. Richard Millechamp, an industrious and skilful apothecary in Bristol. As Guidott recognised that the water had "a Name among the useful Mineral Waters of this Land,"[5] it is of interest to note the method which he took to verify this. A hogshead of the water was, by his direction, evaporated to three or four gallons by Millechamp, who finished it in an evaporating glass and sent the contents, nearly five and a half ounces, to Guidott. Four ounces proved on examination to be "a reddish ferrugineous Earth, somewhat resembling in Colour an Iron Ore,

[1] Part iii., p. 34. [2] A copy of the 1661 "Grant of the Hotwell to the Corporation of Bristol" is given in *Gloucestershire Notes and Queries*, 1884, ii. 223-4. [3] Quoted in Dr. George Randolph's *An Enquiry into the Medicinal Virtues of Bristol-Water*, 1750, p. 13. [4] *Op. cit.*, p. 141. [5] *Op. cit.*, p. 138.

but in Substance very light and friable, with a Mixture of a Lime-stone and somewhat, though much less in Quantity, more white." Guidott then describes his method of dealing with these: "Upon this *Non*-Saline Part, as I call it, to distinguish it from the Saline Part that constitutes the *Lixivium*, [1] being put into a Crucible and calcin'd, I observ'd, That the red Earth was not harder, but more friable, and lost its rusty Colour, becoming more blue; but the white being cold, and mixed with fair Water, did, upon the first Injection, hiss, and afterwards dissolve, leaving the Water white, and a Limy Residence in the Bottom of the Vessel I infused it in; and both white and blue, after Infusion, being dryed again, became very white and Limy. The other Part being Saline, imbibed into a *Lixivium*, I evaporated away to half a Pint, and setting it in a cool Place, found the next Morning it had shot into long small Stiria's [2] to the quantity of ʒiij. The remaining Part of the Liquor that did not shoot, I breath'd away, and had ℥i. of another kind of Salt; [3] so that the Saline Part is here much exceeded by the *non*-Saline, to which it seems to bear proportionably not much more than a 5th Part, and to be contained scarce twelve Grains in a Gallon."[4] From this examination Guidott judged the virtue of the water "to consist of Iron, a Nitro-Sulphureous Salt, and some Lime-stone," and concluded that there was very little of an acid because "neither the cold Water, nor a strong *Lixivium* made of the Salt, will either turn with Galls, or coagulate Milk; neither doth any Thing glebous[5] shew itself among the Shoots." Guidott thought so well of the Hotwell water that he "lamented that so *sanative* a Spring had not a better *Situation*, that so the Water

1 ["**Lixivium.** Lye; water impregnated with alkaline salt, produced from the ashes of vegetables; a liquor which has the power of extraction."—Johnson's *Dictionary*.]

2 ["**Stirious** [*Stiria*, L. an Icicle], hanging, or being in Drops like Icicles."—Bailey's *Dictionary*. Third Edition. 1726.]

3 Of the exact nature of this which he reserved for further examination he says only: "To make it further evident, that this is Lime-stone, after the *non*-Saline part was well calcin'd with a strong Fire, in the Water of which I decocted Sulphur, which it did dissolve, and was precipitated with a fetid smell, both by distill'd Vinegar, Spirit of Vitriol, and Oyl of Tartar, in a considerable Quantity."

4 *Op. cit.*, pp. 139-40.

5 ["**Glebous.** Turfy."—Johnson's *Dictionary*.]

might *expand* itself to the Formation of a *Bath*, which being most temperate in Heat, would be of greater use in all *Tabid*,[1] Emaciating and *Hectic* Diseases, than any other *Bath* in the *Nation* besides."[2] In his *De Thermis Britannicis Tractatus*, published in 1691, Guidott has a chapter, "De Thermis Bristoliensibus," in which he refers to the great value of the water in diabetes.

As the spring issued forth some twenty-six feet below high-water mark, the advantages of its health-giving properties were only obtainable with difficulty. In 1691 a cistern, rising above the level of the highest tide, was constructed to contain the water, but as this came out with much force—at the rate of from forty to sixty gallons in a minute—the arrangement was soon found to be unsatisfactory; and in 1695 a company made an agreement with the Merchant Venturers, who were lords of the manor, for the erection of a pump-room and other facilities for visitors. From this time its popularity grew rapidly, and was promoted by much further medical testimony. A Bristol doctor named John Underhill, practising in College Green, who Latinised his surname into Subtermontanus, published in 1703 a collection of cases[3] which had received benefit from the use of the water, and the records of which had been kept at the Well-house. The epistle dedicatory of his book is addressed "to the Right Worshipful Sir William Lewis, Knight, The Present Mayor, and to the Worshipful the Aldermen and Common-Council of the Well Govern'd City of Bristol," whom Underhill considered to be "an Archetype or Pattern for other Corporations." The water he considered to be "the true *Medela* in that fatal Dejection by Urine and dispiriting, the *Diabetes*, as appears by the *Autography* in the *Hot-Well* House." Underhill's picturesque style is sufficiently represented by his narrative of the classic and historic case of the Bristol baker whose recovery about the end of the seventeenth century from diabetes is recorded by so many of the writers on the Hotwells:

> "Mr. William Gaggs case of *Bristol Castle-Green*, a very Fat man, at his Prime, aged thirty-eight; he was seiz'd with so violent a *Diabeth*, that he made at least three gallons of very sweet Urine with a large quantity of Oil swimming

[1] ["**Tabid.** Wasted by disease; consumptive."—Johnson's *Dictionary*.]

[2] *Op. cit.*, p. 307. [3] *Thermologia Bristoliensis.*

thereon every Night, and could not sleep for either Drinking or Pissing, which in Six Days (his Appetite gone) so run off his Fat and Flesh that he was reduc'd to helpless Skin and Bones, left off by his Physicians (not sparing any Money) and given over by his Wife and Friends for a dead Man (several of his Neighbours then dying of the same Disease, not knowing the Water's Use) resolutely cast himself on God's Mercy and the *Hot-Well* Water (tho' ignorant of its Use) imploring his Friends to support him to the *Hot-Well*, as their last Cast of Kindness, which with Difficulty they perform'd, he fainting every Step and even in drinking the Water, yet to God's Glory and their Astonishment his Strength so came to him every Glass that he made them loose him, pretending to walk, which his Spectators despair'd of and believ'd not, tho' he returned home without Assistance only aided now and then with a Sip of His Holy-water Bottle, his Trusty Friend at a dead lift, the *Hot-Well* Water, which instantly vanquish'd his insatiable Thirst and stopt his Pissing, and so restor'd his deprav'd Appetite, that at his Return Home he eat a large and savoury Meal and by drinking the Water for some-time perfectly attain'd his former State of Health, in all Respects, living many years after."

If this satisfactory result was obtained by a single visit to the spring as seems to be implied in the record, it would have been well calculated to establish the reputation of the Water. Such a recovery would make Lourdes envious. Later historians relate the case with some variations. The intimation is represented by them as coming to the sick man in a dream; but Underhill, who I think is the first narrator of the case, says nothing of this. He gives the names and addresses of several other people who were said to have been cured of diabetes by the Hotwell water, the value of which had so impressed him that he says: "I'll advise such of the Poor that apply themselves to me on the *College-Green Gratis;* and to the rich that desire it *pro Nummulis*,

*Qui caput, & stomachum supponere fontibus audent.*

*Hor. Lib.* 1. *Epist.* 15."

Perhaps his local connections disqualified Underhill from being an impartial judge; in any case it is difficult to take him seriously when he says that the waters "will extinguish the Flame in all *Synochi*, and putrid, if not malignant Fevers: It is *Instar omnium*, the last and only Refuge in Hecticks, and Dyscrasy of Humours. It is of excellent merit, *a Capite usque*

*ad Calcem*, in all Cephalick Cases, and Ataxy of the Spirits, and Palsies, and other Impotencies; and, as to external Uses, it is a trusty Asylum in all left-off incurable Ulcers, Fistula's, and eroding Sores, if not Cancers." [1]

In 1706 Dr. Benjamin Allen instituted some experiments on the qualities of the water, and in *The Natural History of the Mineral-Waters of Great Britain*, published in 1711, he declared it to be "worth trying in Diabetes and where Warmth is useful, and Steel not proper, to make a Constitution firm, as in most Phthises of the Lungs, and wilting [2] Decays; and perhaps in common Hypochondriacal Cases."[3] Allen's analysis produced results which differed from those of Venner and of Guidott. He examined several samples, and in each of them he found that the water gave "a light Golden Yellow and clear with Nutgall, and a bright Claret-red from Logwood boil'd in it. It had so much Acidity, as not to bear Soap; no Liquor disturb'd it by Precipitation or Thickning; it is plainly from a Steam." He goes on to say: "For Matter that inriches it with this Steam; I considered the Rock from under which it proceeds, which is high and large, and this affords Marble in several Places, but near the Spring, a Stone which they burn for Lime; and in some parts a Stone, known by the name of *Bristol* Stone, being a sort of hard Sparr, where the Rock is stain'd with red. The Water not containing a Calcarious Salt, I examin'd the Sparry red part as likely to shew me the Mineral, which with, and without burning, I boil'd in Vinegar, and in the same Distill'd, and extracted a Tincture, as Chymists speak, or inrich'd the Liquor with a taste like white Vitriol, but not very full, tho' plain enough, and which with Nutgall, like that, turn'd a dirty-black: It seems to me to be a Product of Iron and Limestone. The Warmth, since the Rock only lets it out at the Side of it, and is so gentle, may be no Proof of a Sulphur. It appears that the Water contains not any gross Part, or Body of any of the Minerals, or Limestone, and is far enough from Lime VVater, yet

1 Quoted by Randolph, 1750, p. 24.

2 ["**Wilt**, *v.i.* To droop, lose energy.—*v.t.* To render limp or pithless. (Cf. Ger. *welk*, withered.)"—Chambers's *Twentieth Century Dictionary*. 1901.]

3 P. 98.

hath a Vital Effect, plain enough in the Diseases that Lime-Water is us'd in; that the Medicinal part consists in a Gas, or Steam, and Impress from the Mine, whether the Minerals have any considerable Share in the Specifick Effects; whether the Aporrhoe[1] be stronger, according to the Firmness of the Stone; whether a Gas from the Iron be necessary, I leave to further Enquiry."[2]

In 1712 the water received a favourable notice from Sir Robert Atkyns, the historian of Gloucestershire, whose father was Recorder of Bristol from 1662 to 1682. He refers to "the Hot Well, famous for curing divers distempers, especially the diabetes," and to "a very cold stream at Jacob's Well, which is much esteemed for its wholesome waters."[3] In a communication presented to the Royal Society in 1723,[4] Dr. Edward Strother records his "Experiments on Bristol Waters," and concludes that "they are aqueo, salino, alcalino, cretaceo, aluminoso, cupreo, vitriolick, and their effects seem to confirm these experiments."[5]

In a work[6] published in 1725 Dr. J. Wynter recorded what he professed to be a comparison between the waters of Bath and Bristol, the latter of which he believed to consist "principally of *Chalk*, *Lapis Calcarius* and *Calaminaris*, and some lixivial Salt." Much of this is given in a very dogmatic form, as may be seen in the quotations given by Randolph in his *Enquiry*.[7] Wynter's views are summed up by Nott in his 1793 book[8] as looking upon "Bath water as a stimulant, Bristol water as a sedative." Randolph and Nott were both astonished that Wynter considered the water to be of service in dropsy.

Among the visitors about this time were the Duchess of Marlborough, the Duchess of Kent, Lady Diana Spencer, Lady A. Grey, Lord Romney and Sir D. Bulkeley. That the water was in general request is shown by an advertisement which Mr. Latimer, whose three volumes of *Annals of Bristol* are a store-house of accurate information, quotes from the *London Weekly*

1 ["**Aporrhoe**, a flowing down, or issuing from."—Bailey's *Dictionary*, 1726.]
2 P. 42. 3 *The Ancient and Present State of Glocestershire*. Second Edition, 1768, p. 187. 4 Mr. John Latimer's *The Annals of Bristol in the Eighteenth Century*, 1893, p. 139. 5 *Gloucestershire Notes and Queries*, 1884, ii. 240. 6 *Cyclus Metasyncriticus*. 7 Edition 1750, pp. 26-8, 147. 8 *Of the Hotwell Waters, near Bristol*, p. 18.

*Journal* of 30th April, 1726: "Bristol Hot Well water. Fresh from the wells, will be sold and delivered to any part of the town at six shillings per dozen, with the bottles, from Mr. Richard Bristow's, goldsmith, at the Three Bells near Bride Lane, Fleet Street. . . . These bottles are of the largest size, and by the extraordinary favour of the winds arrived but the last week in eight days from Bristol, the common passage being a month or six weeks." Dramatic performances for the amusement of the visitors were given in the Long Room, a building now used for a National School, and one of the representations of "The Beggars' Opera," in 1728, "was attended by 200 persons of the first rank." The actors had come from Bath, where the nobility had presented them with dresses, and it was announced that Mr. Gay would be present at the following representation. The place was, however, not free from calumniators. Richard Savage, who was buried in St. Peter's Church, Bristol, in August, 1743, had not hesitated to revile the city which had befriended him, and addressed it thus:—

"What smiles thy sons must in their foes excite!
Thy sons, to whom all discord is delight;
Thy sons, though crafty, deaf to wisdom's call,
Despising all men and despised by all;
Sons, while thy cliffs a ditch-like river laves,
Rude as thy rocks, and muddy as thy waves,
Of thoughts as narrow as of words immense,
As full of turbulence as void of sense."

And Alexander Pope, who came here for the benefit of his health in 1739, has not much to say in its favour, for he declares "there is no living at the Wells without more conveniences in the winter."[1]

Randolph says[2] that, according to Keir, who wrote in 1739, the water was "a compound of Nitre and *Sea-salt*, intimately united with a *calcarious Earth*;" but Nott,[3] whilst giving Keir credit for his method, looked upon his doctrines and opinions as obsolete. Shebbeare's experiments in 1740, to show that the water consisted "of Alum and Lime-stone, or rather Quick-lime

[1] Letter to Martha Blount. *A Supplementary Volume to the Works of Alexander Pope, Esq.*, 1807, p. 367.

[2] *Op. cit.*, p. 147. [3] *Op. cit.*, p. 18.

slaked," are briefly mentioned by Randolph and Nott, the latter of whom considered Shebbeare's deductions as unpardonable.

Some of the gay doings at the Hotwells in the season of 1743 are quoted by Mr. Latimer. The *Oracle* of 11th June of that year states, that on the previous Wednesday the Earl of Jersey gave a breakfast at the Long Room to 150 persons of high life, and that the Hon. Mr. Ponsonby offered a similar entertainment two days later. Public breakfasts, followed by a dance, were given once or twice weekly, and there were also evening balls, and in imitation of the familiar places of amusement in London, "a piece of ground near the Long Room was opened for evening dances, under the name of the New Vauxhall Gardens, the place being gaily illuminated."

The well-known *Enquiry into the Medicinal Virtues of Bristol-Water* by Dr. George Randolph of Bristol appeared in 1745. Randolph's book was the most ambitious and in many ways the most sensible of all the works that had up to his time dealt with the medical uses of the Hotwell water. He was much struck with "the many notorious Contradictions" of the writers who had preceded him, contradictions sufficiently obvious in the quotations I have already given; and freeing himself from the chemical mystifications of which they were fond, and considering that "Chymical Analysis" is by no means the proper method of procedure, and leaving it to subsequent investigation, looked at the effects of the water from a practical point of view. He doubts Venner's powers of diagnosis, is quite positive that it was not "good against the Spleen,"[1] thinks Guidott was much mistaken about the complaints for which the waters were suitable, ridicules Underhill's records in which the cases were signed by the patients and recognises "what a Medley of Conclusions may arise, when People are left to tell their own Case," and freely criticises all previous advocates of the water. Judged by the medicine of to-day, Randolph may perhaps be condemned with the others in being too easily led to think that many of the patients with serious diseases got well by virtue of the water rather than, as was undoubtedly the case, in spite of it; for his own opinion concerning it was:

[1] This is in opposition to the statement in Fuller's *Worthies*, quoted above.

W. Halfpenny delin. J. Mynde sc.

2—PORTION OF PLAN

OF CLIFTON IN 1746.

LAVARS & C° LITHO. 51. BROAD STREET. BRISTOL.

Impensis S Pye | W. Milton Sculp.

The NORTH-EAST PROSPECT of the HOT-WELL near BRISTOL

Publish'd May y^e 2d 1747 according to Act of Parliament

— ex hoc fonte salutem

"The *first* and principal Virtue is, that of tempering the bad Effects of hot acrimonious Blood; generally preventing, often curing, Inflammations and Hæmorrhage from this Cause; but more especially those of the Kidneys, Womb, and Lungs.

It has, *secondly*, been found of great service in Gleets of both Sexes, and other seminal and uterine Weaknesses; but it is more particularly famous for a Diabetes, in which it is deemed a Specifick.

*Thirdly*, It is a sovereign Remedy in a hectic Fever: It is a notable Preservative against the Stone, not only preventing Gravel from gathering, but powerfully discharging it when gathered; and is a friendly Drink in all inward Ulcers, but more especially those of the urinary Passages." [1]

The value of the spring is then considered under the heading of the several complaints in which it had been found beneficial. Randolph's examination of the water, which he records with great detail on many pages, led him to conclude [2] "that *Bristol* Water, and *Lime-water*, are two different Things," and that the Hotwell water was "a most pure light Water, as free from Recrements [3] as any Water whatsoever; which appears from its carrying uncorrupt round the whole World, and receiving no Alteration, as other Waters will, from hot Climates;" and that its virtues did not "depend upon any Impræegnation of the Water, but upon *impræegnated Air*, which it is very full of, as may be observed at the Pump, by the numerous Air-bubbles that are in it." Dr. Randolph, believing that in great measure he owed his life to this water, offered his book "as a *tabula votiva*, given out in Acknowledgement of the Escape" he had had, and he says in words that may be commended to many an intending author of to-day: "I know the World too well, to commence Author out of Interest; nor am I Fool enough to have any Vanity this Way."

Mr. Latimer records [4] that in 1745 on one of the country journeys to some of the city property "the officials provided themselves with a quart of rum and several gallons of wine, but

[1] Pp. 30-1. The quotations are made from the 1750 edition.

[2] Pp. 157, 173.

[3] ["**Recrement,** any superfluous thing, as dross, scum of mettals, dregs, or dross of perfume, that which is cut or pared away."—Blount's *Glossographia*. Second Edition. 1661.]

[4] *The Annals of Bristol in the Eighteenth Century*, 1893, p. 255.

their stock also included 'six bottles of Hot Well water,' which cost 1s. 6d."

The office of Poet Laureate has been held by many people whose names are well-nigh forgotten. One of these extraordinary creatures, William Whitehead, who received the appointment upon the death of Colley Cibber in 1757, after it had been refused by Gray, had six years before published an allegorical "Hymn to the Nymph of Bristol Spring," belauding the water in the most extravagant manner. It covers eighteen pages in the 1774 edition of his *Plays and Poems*. The author considered the neighbourhood to combine all the beauties of the several English health-resorts which he names.[1] Three years after the date of this poem there is much documentary evidence of the popularity of the Hotwells,[2] which is quoted in detail by Mr. Latimer. Keepers of fashionable shops at London and Bath opened branch establishments here. It is gratifying to our local pride to learn that in a literary direction Bristol set an example to Bath, where a shop was opened for ladies to read the newspapers "as at the Ladies' Tea Room at the Hotwells, at half a crown the season." In Owen's *Observations on the Earths, Rocks, Stones and Minerals for some Miles about Bristol, and on the Nature of the Hot-Well, and on the Virtues of its Water*, 1754, the statement is made that "no price is paid for the water: all the expence that attends the drinking of it, is, that every one, when he goes away, makes a present to the master, and a trifle to be divided amongst the servants." [3] In the same year some interesting light is thrown on social customs,

[1] Addressing Avonia, he says that the poets who seek inspiration in foreign beauty are to be blamed, "Thine is all beauty; every site is thine." The poem was favourably reviewed in the *Gentleman's Magazine*, January, 1751.

[2] Four of Shakspere's plays were performed at the Jacob's Wells playhouse in 1749. (*Gloucestershire Notes and Queries*, 1884, ii. 329.)

[3] P. 134. Of the works which deal with the Hotwells, Owen's book is one of the most interesting I have seen. Amongst other things he says:—

"The people in general are obliging more than in almost any other place I know. Every fine *Sunday* indeed the place is all day long like a fair, vast numbers coming from *Bristol*, and all round, to drink the water; but these go in a back way, and do not interrupt the better sort of company." (Pp. 125-6.) He says that the principal amusements for gentlemen are the river and channel excursions and rides on the Somersetshire side of the river, but of these ladies seldom partake. Their diversions are pretty much confined to the Pump-room for we are told that "Elizabeth Trinder, from the Lebeck's Head Tavern, Bath, has opened a house at the Hotwells for the reception of company as a tavern or eating-house. An ordinary everyday at three o'clock, at half-a-crown a head . . . the house being the first of the kind attempted here." The house which was called 'The Lebeck' still bears the name, but during recent years it has been used by the Government as a Recruiting Office. A year later the Hotwell water was affected by the Lisbon earthquake. It became discoloured to such an extent that people thought the end of the world was upon them, and "flew to the churches, where prayers were offered to avert the apparent approach of their destruction."

In 1756 Dr. Charles Lucas published *An Essay on Waters*, in which is a section "Of the Baths, or Hot-well Waters, near Bristol." After a description of the charming scenery of the neighbourhood, he gives an elaborate comparative analysis of the three springs which had achieved a reputation more than local. These he calls the Old Hot-well, the New Hot-well, and the Mill-spring.[1] Mr. Latimer traces [2] the fortunes of the "new" Hotwell spring, at which John Wesley, in 1754, was a visitor because he preferred it to the "noise and hurry" of the older spring. The Mill-spring on the other side of the river furnished a cold supply, and was doubtless that copious flow which has been recently diverted. Lucas was led to his analytical view because there had been "great and irreconcileable diversities of opinion about the nature of the Bristol and the Long-room. But some take great delight in riding upon Durdham-Down, and the best lady attending the Hot-well will not refuse riding behind a man, for such is the custom of the country, and numbers of what they call double horses are kept for that purpose. Owen says that at Bristol it was seldom necessary to drink the water under medical advice, as it was at Bath. Plainly speaking about doctors, he adds: "It were well if those gentlemens fees were all the ill that sometimes attends their officious service." (Pp. 135-6.) For medical men the fashionable life at the Hotwells does not seem to have been a very good thing: "Excepting for now and then a prescription for a bottle of drops to an old lady, or some salts to a fine gentleman who wants to soften his complection, the doctor seldom picks up many guineas." (P. 136.)

[1] The sites of all these are shown on the 1746 plan.

[2] *Op. cit.*, pp. 265, 463-4.

Hot-well water." His labours were intended "to compose and solve these doubts," and he introduces his conclusions by saying "Let such nameless authors rest in peace: let us come to facts." These facts he considered were sufficient to justify him in saying "A summary of the impregnation of these three waters, is this: first, the common base of the composition, simple water; by exhalation from decomposing pyrites, at a certain distance, impregnated with some of the grosser, but more of the subtilised, universal acid, and also by those, heated to the degree, set forth. Secondly, water thus charged, meeting with an alcaline and calcarious earth, with some proportion of the mercurial principle, it is not difficult to conceive how the impregnation, we find, should be produced. 1. The grosser, with the volatile acid, meeting with an alcaline earth, or the mineral alcali, give the vitriolate salt, discovered in these waters. 2. These acids, with the mercurial principle, and the alcaline base, constitute the sea salt, observed in them. And, 3, these acids, meeting with, and dissolving, absorbent earths, must yield a calcarious earth and a selenite upon evaporation. Agreeable to this theory it is, that these waters appear, by our experiments, to be thus impregnated. And from these principles, their principal virtues are to be adduced."[1] Except in conditions "such as hectics, diabetes, &c." where a water of the particular temperature of the old spring was necessary he preferred "the simple neglected Mill spring."

*A Methodical Synopsis of Mineral Waters* is a portly quarto of 660 pages which Dr. John Rutty issued in 1757. He deals with the Bristol water mostly from the point of view of analysis for the purposes of which he used samples from the stock which was on sale in Dublin. He records his results with great exactness, and gives also summaries of the methods employed by other experimenters. The water was not "the product of a calcination by a subterraneous fire." Rutty quotes various writers on the use of the water in disease.

In 1758 Dr. A. Sutherland of Bath published a work[2] on the Bristol water, in which he is very severe on Randolph, who in

[1] Part III., p. 366.

[2] *The Nature and Qualities of Bristol Water.*

his opinion was unduly depreciatory of the records upon which Underhill based his observations; he condemns Randolph's "errors and absurdities" and thinks him entitled to the advice contained in the lines—

> "Launch not beyond your depth, but be *discreet*,
> Mark well the point where Sense and Dulness meet."

Dissatisfied with all previous analyses, he subjected the water to a searching independent examination with the assistance of his friend, Dr. Baylies,[1] the "Master of a neat experimental Apparatus, as well as a cabinet of the *Materia Medica*, which might claim a place in any University, and which the Owner not only possesses, but also understands." These experiments are recorded at great length and the sum of them is given in these words: "1. That those who have deemed *Bristol Water* to be a simple elementary Fluid, have founded their opinion merely upon ignorance. 2. That those who have charged them with *Iron*, *Nitre*, *Alum*, *Vitriol*, *Sulphur*, *Lime*, &c., have either grounded their Opinions without Experiment, or have erred in their Analysis," and he affirms positively that the Bristol Waters are efficacious because "they contain: 1. The Spirit. 2. The pure Element. 3. A Vitriolic Acid. 4. A Marine Acid. 5. A Neutral Salt. 6. An Absorbent Earth."[2] Sutherland then considers these separately, defining the first as "its subtile *Æther*." He then says the greatest part of the virtue of some medicinal waters is owing to the "pure element." In this perhaps most modern observers would agree with him. But then, apparently quoting from Baylies's observations, he declares that the chief, perhaps the sole, effect of the Water is due to the "Volatile Vitriolic Acid," an exhalation which is said to be continually breathing up from the Vitriol Stone which abounds in the bowels of the earth. The "Marine Acid," more easily recognised as common sea salt, is said to blend with the vitriolic acid, and the two together "remarkably resist putre-

[1] An interesting insight into Bath medical life is given in Dr. Baylies *Practical Reflections on the Uses and Abuses of Bath Waters . . . to which is added . . . A Narrative of Facts relative to the Physical Confederacy in Bath.* 1757.

[2] *Op. cit.* A New Edition. 1788. Pp. 63-4.

[3] *Op. cit.*, pp. 72-96.

faction, and subdue those fevers which are the consequence of Pus absorbed and carried through the *circulation*." The "simple Calcarious Earth" which was found was said to render the Waters safe and wholesome, and to give them the "property of correcting Acidities in the first passages," and this was sufficient to account "for those cures, which they daily performed in obstinate Fluxes, Gleets, and Female Weaknesses." Sutherland was careful not to promise too much from the use of the waters; "he who expects to find relief at this spring, must submit to a long course of attendance, he must arm himself with a stock of patience and perseverance. To these he must join temperance of every kind."[1] The good results were in constant danger. "The practice of drinking tea twice a day, or even once, is absurd, it answers only one purpose, that of rendering the virtues of the Waters less effectual."[2] Sutherland believed that "infection" and "translation of morbific matter" often produced consumption, a disease in which the water had proved to be a remedy both easy and effectual, and he had found it useful not only in hæmoptysis but in many other hemorrhages. As a cure for diabetes, "none bids so fair for the name of a *Specific* as Bristol Water,"[3] and he narrates how each of the constituents into which he had resolved it played its part in performing this wonder. Sutherland ends his 151 pages with a long list of other diseases, in almost all of which "it will relieve, where it cannot cure." Sutherland came to the conclusion "that as Mineral Waters in general, so Bristol Waters in particular are of such Efficacy, for the *preservation* of health, as well as he *cure* of Diseases, as in the highest degree, to exceed all *Shop Remedies*, and that they approach the nearest in nature to what has vainly been searched after, An Universal Medicine." In reference to the general attraction of the place, Sutherland states that "provisions of all sorts, are to be had in plenty, during the Summer, which is the season allotted, by custom, for drinking these Waters. Garden Stuff is early, and excellent. There are Lodgings near the Wells, convenient for such as are real Invalids; there are magnificent Lodgings in the beautiful village of Clifton, on the top of the hill, for

[1] *Op. cit.*, p. 122. [2] *Op. cit.*, p. 123. [3] *Op. cit.*, p. 142.

such as have carriages, and whose lungs can bear a keener air. There are *Balls* twice a week, and *Card-playing* every night." [1]

4.—*Clifton and Redland Roads, from MS. plan about 1759.*

The impartiality of this Bath physician[2] is shown by the plea he urges for improvements in order to make the advantages of the place more accessible to visitors.

[1] *Op. cit.*, p. 17.

[2] Those who are curious to see the style of criticism in vogue about the middle of the eighteenth century should turn to the *Cursory Remarks* of Lucas when considering Sutherland's method of investigating the Bath and Bristol waters. Continental opinion about doctors assumed an anti-British form, and Lucas says that the scandalous practices charged upon many practitioners in Bath and Bristol were only too true, and he gives a long list of the devices which were used to obtain and retain patients. He designates Sutherland as "a man of parts and letters, who has written a larger volume than any of his Contemporaries, which at Bath is the sure test of his being the greatest man,"

There was still great demand for the water from a distance. It was kept on sale, wholesale and retail, by Fairly Jones at the Golden Wheat Sheaf in Tavistock Street, Covent Garden, who stated that the stock was "constantly fresh and certify'd by the Proprietors of the Well." A reproduction of the label is here given, taken from a book by Dr. Diederick W. Linden, which reached a third edition in 1759.[1]

Dr. Linden, who very kindly kept a watchful eye over his patients who came to the Hotwells, where he spent a portion of the summer with them, remonstrated in the *Gentleman's Magazine* in August, 1761, against the evil effects of a lead-smelting house which five years before had been established on the other side of the river nearly opposite the Pump Room of what Linden calls "the second medicinal spring in the kingdom." Linden was considered so great an authority on hydropathy that some of his friends celebrated his attainments in verse which his modesty did not preclude him from printing in his book. Amongst the fulsome things which have been said or written about individuals, this extract will unanimously be accorded a bad eminence :—

"And when no more these *Earthly Streams* afford
That Health to him, to others they restor'd ;
When *Galen's* Sons shall his sad Loss deplore ;
His Skill, in Consultations, reap no more :
When the *Castalian* Nymphs, in mournful Tale,
The *Universal Friend's* Departure wail ;
*Seraphs*, to deck them, and emblaze his Fame,
Shall o'er the Skies bespangle LINDEN'S Name ;
In glittering Characters, it there shall shine,
A *Constellation* in the *Watry Sign :*
While, bath'd in Bliss, he wafts at full Content,
In *Heavenly Streams*, above the Firmament."

adding that "the learned gentleman, from pure patriot principles, contends for Sulphur in Bath Waters ; well knowing, that since *Solomon* the son of *David* sat upon the throne of Britain, there never was a time, in which there was so great a necessity of having England plentifully stored with Brimstone, as those happy days, in which our Author florishes." In reference to Sutherland's views about the Bristol water Lucas says very little, pouring out the vials of his wrath upon Sutherland for his opinions on the springs of Bath, "where the Faculty arrogate to themselves more infallibility than the gentlemen of the profession do any where else." (Pp. 5-9.)

[1] The first edition appeared in 1748.

This was written by William Oldys, who was Lord Oxford's librarian. Linden appears with a very thin disguise in Dr. Smollett's *Humphrey Clinker*, in which the social life of the Hotwells is viewed from very different standpoints; a good deal of the scenes of Miss Burney's *Evelina* is also laid at the Hotwells.

5.—*A View of St. Vincent's Rocks about 1760.*

In 1766 the Duke of York came here to get the benefit of the waters. His visit was commemorated by attaching his name to a well-known inn at the Hotwells. Mr. Latimer says that the author of *The Beauties of England*, which was published in 1767, noticed when in Bristol that the water was not only drunk on the spot at the Pump Room but every morning cried in the streets like milk, and the *St. James's Chronicle* of 1st July, 1769, said: "We hear from the Hot Wells that there is a good deal of very good company already; seldom less than 200 at the public breakfasts, with cotillons, and fuller balls than were last year at the height of the season, which is generally about the third week in July." The place was resorted to not only in the usual season; persons of independent fortune had on account of its many attractions either purchased or taken houses in order to live there winter and summer. "The inhabitants met twice a week last winter to drink tea and play at cards, which encreased its sociability." In 1771 "Stage-coaches began to ply between Bristol and the Hotwells, at sixpenny fares."[1]

[1] Evans's *A Chronological Outline of the History of Bristol*, 1824.

Dr. John Fothergill[1] had seen "many persons recover from pulmonary diseases after drinking the Bristol water, whose cure seemed to be doubtful from any other process." After allowance had been made for better air, change of situation and of objects, and regular mode of living in which fresh air had a considerable part, it still seemed to him "that the water drank fresh at the pump, actually contains principles conducive to the recovery of patients affected with phthisical complaints," and he considered it to be "of signal benefit to consumptive patients," and that when change of air is recommended, Bristol should first of all claim attention.

Dr. John Elliot, of London, in 1781 gave a long list[2] of the disorders in which the Bristol water had been recommended. He says that "the usual method of drinking the water is a glass or two before breakfast, and about five in the afternoon. The next day three glasses before breakfast, and as many in the afternoon; and this is to be continued during the patient's stay at the Wells. A quarter or half an hour is allowed between each glass. A course of these waters requires no preparation further than to empty the bowels by some gentle purge; and if heat or fever requires, to take away a few ounces of blood. Costiveness, however, should be avoided during the course."

In 1785 the exportation of the water took place with the authority of the Merchant Venturers, and every bottle sent out bore the impression of a seal which had been engraved for the purpose. There was at that time, and for many years after, much to occupy and divert the gay company by which the place was thronged. Its reputation in 1789 may be gathered from a description given by Dr. Andrew Carrick, who says: "The Hotwells during summer was one of the best-frequented and most crowded watering-places in the kingdom. Scores of the first nobility were to be found there every season, and such a crowd of invalids of all ranks resorted to the waters that it was often difficult

[1] "Further Remarks on the Treatment of Consumption, &c." *Medical Observations and Inquiries.* By a Society of Physicians in London. Vol. v., 1776, p. 345.

[2] *An Account of the Nature and Medicinal Virtues of the Principal Mineral Waters of Great Britain and Ireland,* p. 114.

6.—" *View of Bristol Hot-Well*," *about 1770.*

for them to provide themselves with any sort of lodgings. About that period a considerable number of lodging letters had in the course of a few years realized very handsome fortunes, without any complaint of extortionate exactions. Three extensive taverns were constantly full, and two spacious ballrooms were profitably kept open. There was a well-attended ball, a public breakfast, and a promenade every week, and often twice a week. The pump-room was all day long the resort of invalids, who left with the keeper of the well many hundreds a year in voluntary donations, and from twelve to two o'clock was generally so crowded that there was often some difficulty in getting up to drink the water. The walk adjoining was in the meantime filled with fashionable company, to whom the sublime scenery of the cliffs was enlivened by the sounds of a band of music. The downs and all the avenues to the Hotwells were filled with strings of carriages, and with parties on horseback and on foot."[1] In *Shiercliff's Bristol and Hotwell Guide*, published in 1789, we learn that "no persons need be at a loss for amusement during their residence at the Hotwell." The river excursions are described and in connection with them it is said "the effect of the music on the water, especially when re-echoed from the rocks, is enchanting, and inspires the most agreeable sensations." The Rev. George Heath, the compiler of Matthews's *New History of Bristol, or Complete Guide and Bristol Directory for the Year, 1793-4*,[2] who was greatly indebted to Shiercliff for descriptions which he obviously paraphrased, adds that "many ladies and gentlemen, cross the River at *Rownham* Ferry and walk to the sweet and wholesome village of *Ashton* to eat strawberries or rasberries with cream: a delicious and salutary repast." After the public breakfasts there were cotillons and country dances, and for these and the balls, which were held every Tuesday, there was a Master of Ceremonies.

The reputation of the Hotwell water was at this time so great as to become itself a danger, for as a last resource consumptive

[1] *The Annals of Bristol in the Nineteenth Century*, 1887, p. 71.

[2] A facsimile edition of this was published by Messrs. John Wright & Co. in 1898.

patients in a dying state were often sent here. Echoing a warning note first sounded by Owen in 1754, in reference to this practice which was so likely to affect injuriously the credit of the place, *Shiercliff's Guide* says: "We do not wish to cast any reflection on the gentlemen of the faculty whose advice they have consulted, but we are afraid it is too often a practice with them not to part with a patient, whilst they have the least probability of success; when they find their art ineffectual, and the case desperate, then and not till then, the physician consigns his patient to the Bristol Hotwell to try the effect of the water, by which he avoids the imputation of their dying under his hands."

*(To be continued.)*

---

### NOTES ON THE ILLUSTRATIONS.

For enabling me to give some of the illustrations which accompany this paper I am indebted to the courtesy of the authorities of the Bristol Museum and Library, to Mr. J. W. Arrowsmith (the publisher of *Bristol: Past and Present*), to Mr. A. E. Hudd (the Honorary Secretary of the Clifton Antiquarian Club), and to Mr. J. E. Pritchard.

1 (Inset). This is a representation, about the year 1735, of the pump-room that was erected in 1696, and of the buildings in the immediate neighbourhood. Barrett in his list (*History of Bristol* [1789], p. 112) does not mention this view, although he refers to one of the Drawbridge by Halfpenny and Mynde, and one of the Infirmary by Halfpenny. Neither in designer nor in engraver was this illustration fortunate. William Halfpenny was a London "architect and carpenter," (see *Dictionary of National Biography*,) indications of whose profession and work can be seen in the formal outlines of the drawing. J. Mynde was an engraver of small reputation whose work was principally done for the booksellers. (See Bryan's *Dictionary of Painters and Engravers*.) Beginning in 1722, William Halfpenny published several books on Building and Architecture. For some of these he had the help of John Halfpenny, who "is said to have built 1744, Coopers' Hall and 1789—94, S. Paul's Church, Portland Square, both at Bristol" (*Dictionary of Architecture*). The dates render this statement somewhat improbable, and in reference to the Church, Evans says (*A Chronological Outline of the History of Bristol*, 1824) that "the architect and builder was Daniel Hague."

2 (Inset). In 1746 the Merchant Venturers' Society had a coloured plan made to show their Clifton property. A copy that was made for one of the Goldney family appears in a reduced form in vol. v. of the *Proceedings of the Clifton Antiquarian Club*, with a description by Mr. John Latimer. The illustration here given is a portion of this (slightly enlarged), but without the colouring of the copy from which it was taken. The places can easily be made out by noting one or two landmarks. Clifton Church is shown by *Z* O, and F represents nearly the situation of the present post-office. Clifton Vale is indicated by the Mead Lane. The position of some of the lettering has been slightly altered in order to bring it within the portion reproduced.

3 (Inset). Whilst No. 1 is almost diagrammatic, this, which gives much the same view in 1747, probably errs in being too picturesque. It was most likely drawn by Milton, who was no doubt the William Milton mentioned by Redgrave (*Dictionary of Artists*). Barrett (*loc. cit.*) refers to a view of the Infirmary drawn and engraved by Milton. That which is now known as the Colonnade was not then in existence. The bearer of the cost of the original engraving was a Bristol surgeon, the line of whose practice was referred to by Chatterton (see Greig Smith's *Abdominal Surgery*, section on "Supra-pubic Cystotomy."):

> "The home-bred documents of old Sam. Pye
> Were standing rules to treat their buboes by."

In 1724 Pye published some *Some Observations on the several Methods of Lithotomy.*

The prints from which Nos. 1 and 3 were taken are in the Museum and Library.

4 (p. 19). This plan comes from *Bristol: Past and Present* (III. 194), and represents the principal Clifton roads about the year 1759. With the exception of the roads which led to Jacob's Wells, one only is shown from the higher ground to the Hotwells, going somewhat on the line of Beaufort Buildings and leading to Granby Hill. That which is marked on the 1746 plan as the Mead Lane was probably not of sufficient importance to be called a road and was no doubt unfitted for wheel traffic.

5 (p. 21). This is also from *Bristol: Past and Present* (III. 225). It is a reduced illustration from a picture in Barrett (*op. cit.*, p. 92). The low building on the right is Mr. Warren's house, the site of which is also indicated on the 1746 plan, occupying ground somewhere near the present Clift House before the New Cut was made.

6 (p. 23). In this view, taken direct from Barrett, the Spa buildings are shown between 1766 and 1789, after the Colonnade had been built. The "colonade" referred to by Nott (*Of the Hotwell Waters near Bristol*, 1793, p. 91) was probably a piazza connected with the pump-room, indications of which are to be seen in this view. It also gives the snuff-mill which was erected, about 1766, on the site of the present Observatory. The snuff-mill was burned down in 1777. The Observatory dates from 1828. The name of John Doddrell appears in the Bristol Directories down to 1813 as carrying on his work as engraver in Avon Street, Great Gardens.

All these views of the Hotwells give a very inadequate idea of the extent of the accommodation that existed for visitors, both pleasure-taking and invalid. Barrett says (p. 94) "the buildings lately erected there give it more the appearance of a large town than of lodgings for the sick alone, and have so increased of late as to join the Hotwells quite to Bristol, by an uninterrupted chain of houses."

## Figures and Tables

**1. f1:**
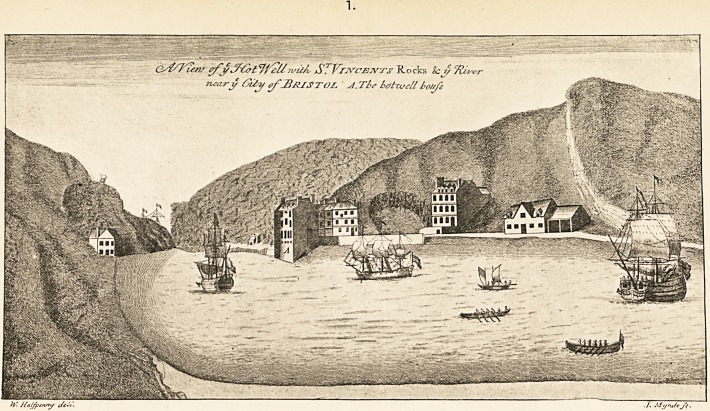


**2 f2:**
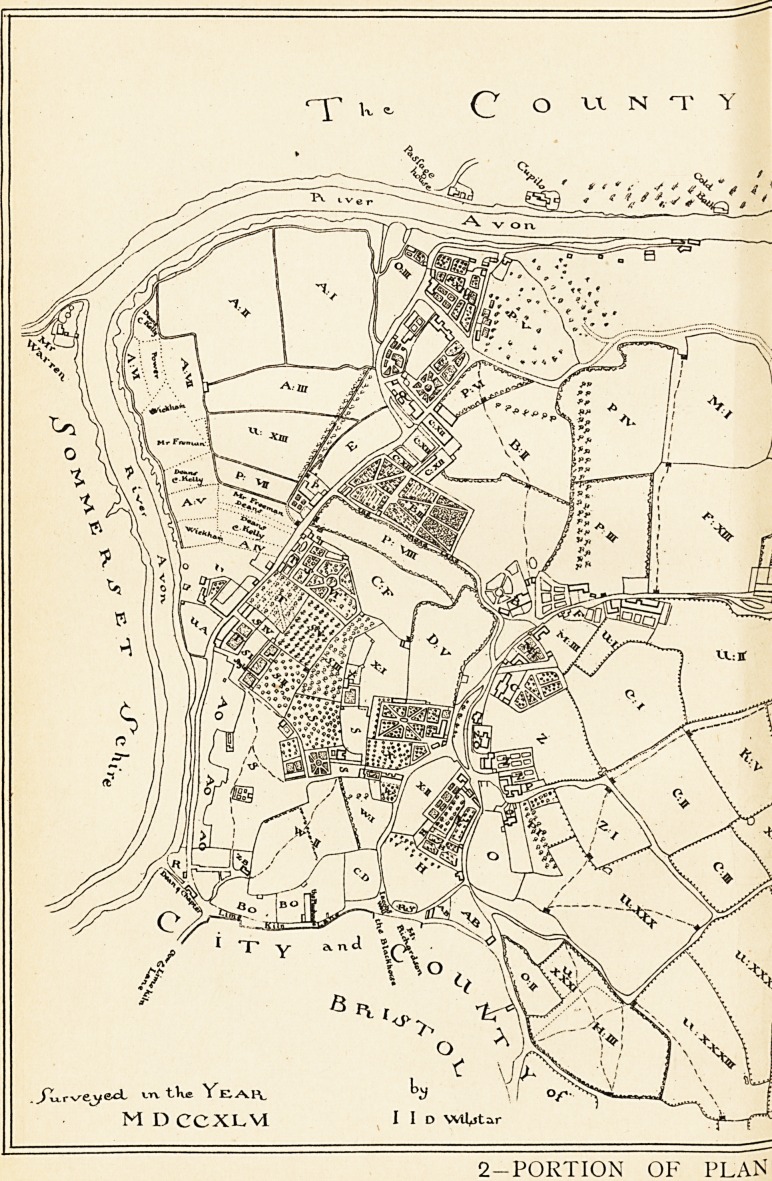


**Figure f3:**
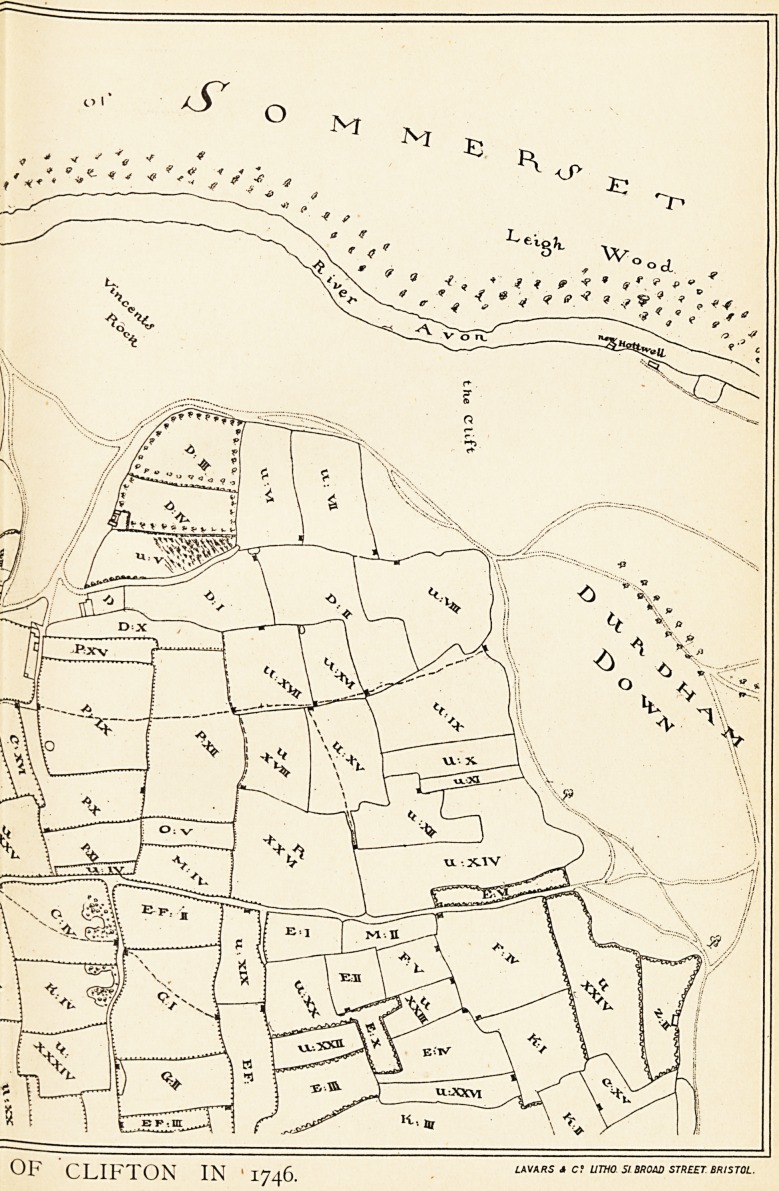


**3. f4:**
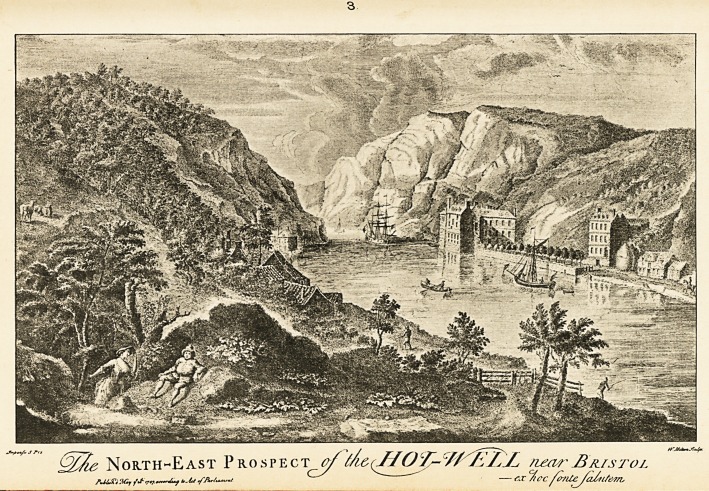


**4. f5:**
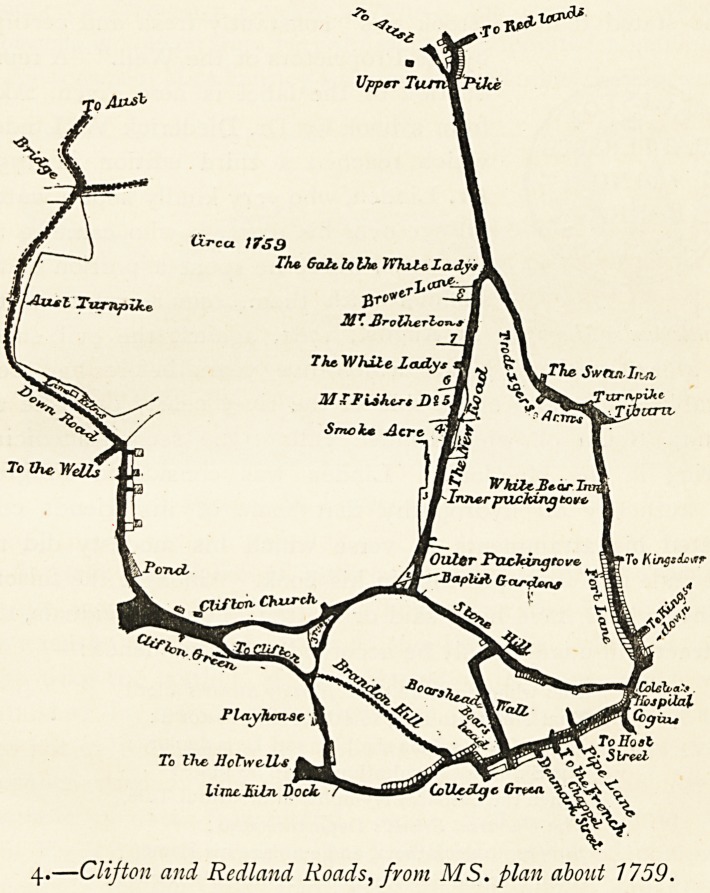


**Figure f6:**
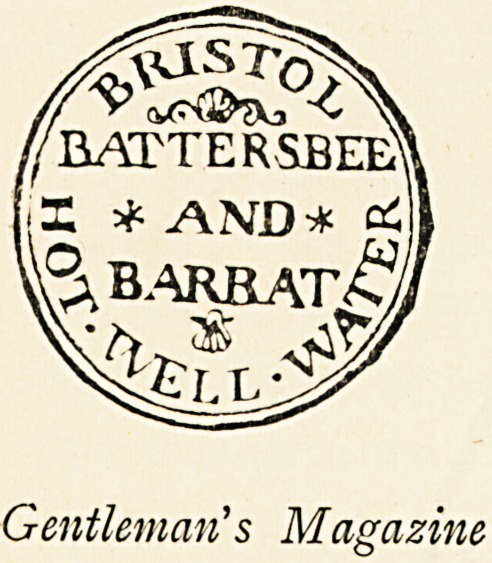


**5. f7:**
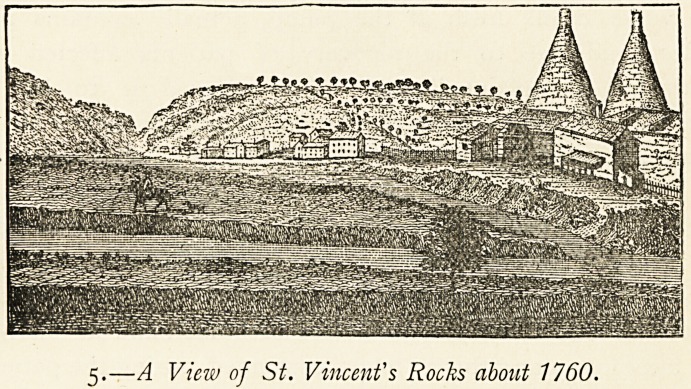


**6. f8:**